# Association and mediation between educational attainment and respiratory diseases: a Mendelian randomization study

**DOI:** 10.1186/s12931-024-02722-4

**Published:** 2024-03-06

**Authors:** Guohui Lan, Mengying Xie, Jieli Lan, Zelin Huang, Xiaowei Xie, Mengdan Liang, Zhehui Chen, Xiannuan Jiang, Xiaoli Lu, Xiaoying Ye, Tingting Xu, Yiming Zeng, Xiaoxu Xie

**Affiliations:** 1 Department of Epidemiology and Health Statistics, School of Public Health, Fujian Medical University, Fuzhou, China; 2 The Second Clinical Medical School, Nanchang University, Nanchang, China; 3 Clinical Research Unit, The Second Affiliated Hospital, Fujian Medical University, Quanzhou, China; 4 The First Clinical Medical School, Shanxi Medical University, Taiyuan, China; 5 Department of Pulmonary and Critical Care Medicine, Center of Respiratory Medicine of Fujian Province, The Second Affiliated Hospital of Fujian Medical University, Quanzhou, China

**Keywords:** Education, Lung function, Lung cancer, Asthma, Mendelian randomization

## Abstract

**Background:**

Respiratory diseases are a major health burden, and educational inequalities may influence disease prevalence. We aim to evaluate the causal link between educational attainment and respiratory disease, and to determine the mediating influence of several known modifiable risk factors.

**Methods:**

We conducted a two-step, two-sample Mendelian randomization (MR) analysis using summary statistics from genome-wide association studies (GWAS) and single nucleotide polymorphisms (SNPs) as instrumental variables for educational attainment and respiratory diseases. Additionally, we performed a multivariable MR analysis to estimate the direct causal effect of each exposure variable included in the analysis on the outcome, conditional on the other exposure variables included in the model. The mediating roles of body mass index (BMI), physical activity, and smoking were also assessed.

**Findings:**

MR analyses provide evidence of genetically predicted educational attainment on the risk of FEV1 (β = 0.10, 95% CI 0.06, 0.14), FVC (β = 0.12, 95% CI 0.07, 0.16), FEV1/FVC (β = − 0.005, 95% CI − 0.05, 0.04), lung cancer (OR = 0.54, 95% CI 0.45, 0.65) and asthma (OR = 0.86, 95% CI 0.78, 0.94). Multivariable MR dicated the effect of educational attainment on FEV1 (β = 0.10, 95% CI 0.04, 0.16), FVC (β = 0.07, 95% CI 0.01, 0.12), FEV1/FVC (β = 0.07, 95% CI 0.01, 0.01), lung cancer (OR = 0.55, 95% CI 0.42, 0.71) and asthma (OR = 0.88, 95% CI 0.78, 0.99) persisted after adjusting BMI and cigarettes per day. Of the 23 potential risk factors, BMI, smoking may partially mediate the relationship between education and lung disease.

**Conclusion:**

High levels of educational attainment have a potential causal protective effect on respiratory diseases. Reducing smoking and adiposity may be a target for the prevention of respiratory diseases attributable to low educational attainment.

**Supplementary Information:**

The online version contains supplementary material available at 10.1186/s12931-024-02722-4.

## Key messages

### What is already known on this topic

Several observational studies have revealed that people with a higher education attainment is associated with a lower risk of developing respiratory diseases. However, observational studies are susceptible to reverse causation and confounding factors. Also, the role of genetic factors in the study remains unknown.

### What this study adds

In this study, by leveraging data from the recently published genome-wide association studies, we found a significant genetic correlation between educational attainment and respiratory disease. We further confirmed that the causal relationship between educational attainment and respiratory disease is partially mediated by smoking and obesity.

### How this study might affect research, practice or policy

Our study highlights the importance of early detection and prevention of respiratory disease, including lung function, lung cancer and asthma, amongst low education group. Moreover, our findings might provide new understandings for the mechanisms associated with educational attainment and respiratory disease.

## Introduction

Deaths from chronic respiratory diseases constituted 7% of all deaths globally in 2019, with the prevalent diseases including chronic obstructive pulmonary disease (COPD), asthma, and lung cancer [1]. Identifying potential risk factors is crucial to safeguarding public health and preventing the emergence of diseases. Lung function is an important predictor of quality of life and longevity [2].

Socioeconomic disparities in health have been documented. Individuals with lower socioeconomic status have higher mortality and morbidity risks compared to individuals with higher socioeconomic status. There has been research into the impact of socioeconomic factors on health outcomes [3–7]. Among many socio-economic indicators, educational attainment (EA) has been identified as a social determinant of health through various mechanisms, such as neurodevelopment, health behavior, and health literacy [8].

Several studies have examined the association between EA and respiratory diseases. Previous studies have employed cross-sectional designs to investigate the complex relationship between EA and lung function [9] and lung cancer [10]. There have been several studies that have examined the effects of education on pulmonary health, and they have also identified potential mechanisms or mediators that may explain these effects. The finding suggest that this association may be mediated by some modifiable factors related to both exposure and outcome, such as BMI, physical activity and smoking. Nevertheless, these studies were observational in nature and thus prone to methodological limitations, including confounding and reverse causality, as well as failure to consider mediating factors. Therefore, the relationship between EA and respiratory disease, as well as lung function is unclear.

Mendelian randomization (MR) is a method for inferring causal relationships based on genetics, utilizing single-nucleotide polymorphism (SNP) as a surrogate of exposure, evaluating observed data and correlating causal relationships through a statistical relationship between genotypes and phenotypes [11]. Under several assumptions, an MR study should produce results which avoid the potential biases associated with observational studies, such as confounding, reverse causation, and measurement errors, which are common in observational studies. Multivariable Mendelian randomization (MVMR) is a rapidly evolving analytical method that estimates the effect of each exposure variable on the model results while also considering the effects of other exposure variables that may affect the model results [12]. This method is based on Mendelian inheritance law, randomly grouping multiple variables simultaneously to create a random distribution of variables between the groups, thereby enhancing the reliability and accuracy of the experiment.

The correlation between EA and asthma, COPD, and lung cancer has been reported in previous studies [9, 10, 13], the causal mechanism is not clear and these did not assess mediation by modifiable factors. In addition, recent GWAS on compared with the previously reported GWAS, a newly published GWAS for EA comes from a large sample of population data, and the results are more accurate. Therefore, using the latest GWAS data, it is possible to update the results of studies on the relationship between EA and respiratory diseases, in order to better understand this association. Potential confounding factors were also included in the MVMR analysis to control for their effects and obtain more accurate estimates of the direct causal effect of each exposure on the outcome.

In this report, the MR method was used to assess the causal association between EA and lung function, asthma and lung cancer, and a two-step Mendelian randomization was used to assess its mediated proportion in association for 23 potential mediating factors. Ultimately these causal conclusions will support the development of prevention policies.

## Methods

### Study design

In Mendelian randomization research, genetic information is usually used as an instrumental variable (IV) due to their random distribution in humans and robust associations with the exposure and outcome variables being investigated. EA was assessed causally associated with lung cancer, FEV1, FVC, FEV1/FVC and asthma using two-step Mendelian randomization analysis. All GWAS summary statistics were gathered from a public GWAS website (https://gwas.mrcieu.ac.uk/) for the purposes of these analyses. Data summarized from GWAS are presented in Table 1.

**Table 1:** Overview of GWAS data used in multivariable Mendelian randomization (MVMR)

Phenotype	Number of participants	Number of SNP	Consortium	Author	PubMed ID	GWAS ID
Year of schooling	766,345	10,101,242	SSGAC	Lee et al.	30038396	ieu-a-1239
FEV1	321,047	19,641,887	–	Shrine N	30804560	ebi-a-GCST007432
FVC						ebi-a-GCST007429
FEV1/FVC						ebi-a-GCST007431
Lung cancer	27,209	8,945,893	ILCCO	Wang Y et al.	24880342	ieu-a-966
Asthma	408,442	34,551,291	–	Valette K et al.	34103634	ebi-a-GCST90014325
BMI	681,275	2,336,260	GIANT	Loic Yengo et al.	30124842	ieu-b-40
physical activity	215,127	15,538,177	–	Tyrrell J et al.	33563987	ebi-a-GCST90012791
Cigarettes per Day	337,334	11,913,712	GSCAN	Liu, M et al.	30643251	ieu-b-25

*SSGAC* Social Science Genetic Association Consortium, *ILCCO* International Lung Cancer Consortium, *GIANT* Genetic Investigation of Anthropometric Traits, *GSCAN* GWAS & Sequencing Consortium of Alcohol and Nicotine use, *EA* educational attainment, *BMI* body mass index, *FEV1* forced expiratory volume in one second, *FVC* forced vital capacity, *FEV1/FVC* forced expiratory volume in one second / forced vital capacity

### Education attainment

The genetic instruments for EA were selected from a meta-analysis comprising 71 GWAS discovery cohorts that included 1, 131, 881 European ancestral participants. To facilitate the classification and conversion of educational levels into standardized units for better cross-country and cross-regional comparisons, the International Standard Classification of Education (ISCED) 2011 was employed, utilizing 4.2 years of education as the unit within the educational systems of the UK and the US. In a study with the identifier ieu-a-1239, conducted on a sample of more than 1.1 million individuals, a genetic association analysis of EA was performed. This analysis identified 1271 independent SNPs that exhibited significant associations with EA [14].

### Outcome-respiratory disease

The outcomes used in this study were lung function indicators and related lung diseases (lung cancer and asthma).

#### Lung function

We selected respiratory function indicators to assess lung function in 400,102 European ancestry individuals. We identified 139 new signals related to lung function, including forced expiratory volume in one second (FEV1), lower forced vital capacity (FVC), and the FEV1-to-FVC ratio [15]. ID: ebi-a-GCST007431 (FEV1/FVC), ebi-a-GCST007429 (FVC), ebi-a-GCST007432 (FEV1).

#### Lung cancer

The International Lung Cancer Consortium (ILCCO) conducted a GWAS analysis on lung cancer and identified 259 SNPs (with a significance level of P < 5 × 10^–8^) in a study involving 11,348 lung cancer cases and 15,861 controls [16]. ID: ieu-a-966.

#### Asthma

Valette et al.[17] used genetic instruments from the UK Biobank in a study that employed a broad definition of asthma. The study included 56,167 asthma cases and 352,255 controls. ID: ebi-a-GCST90014325.

### Mediators

Based on our review of the literature, we selected 23 candidate mediators of modifiable risk factors (please refer to Additional file 1: Fig. S1 in the supplementary materials for an overview of the process of identifying the candidate mediators). The mediators involved in the relationship between EA and respiratory disease were selected based on the following criteria for inclusion in the analysis: (1) Exposure and mediating factors are related in a causal way; (2) There was an association between mediating factors and outcomes, whether or not exposure factors were corrected. Ultimately, we identified three risk factors that met the criteria, including BMI [18], physical activity [19] and cigarettes per day [20], were included in the mediation analysis to assess the role of mediation between EA and lung function, lung cancer, or asthma. ID: ieu-b-40 (BMI), ebi-a-GCST90012791 (physical activity), ieu-b-25 (cigarettes per day).

### SNP selection

To conduct Mendelian randomization, we selected the instrumental variables (IVs) as follows. Firstly, we selected SNPs that were significantly associated with educational attainment for each MR analysis, excluding genetic instruments with P values greater than 5 × 10^–8^ in relation to the exposure. Secondly, as part of the MR analysis, we utilized independent SNPs as genetic instruments when genetic associations were identified for both the exposure and the outcome of interest. Then, the clumping process (*r*^2^ < 0.001 within 10,000-kb windows) was employed to determine whether the included SNPs are in linkage disequilibrium (LD). If no SNPs related to exposure were identified in the results, we did not utilize proxy SNPs. Finally, to ensure that there was no direct correlation between the instrumental variables used in the analysis and the outcome, excluding genetic instruments with P values < 5 × 10^–8^ in relation to the outcome.

### Statistical analysis

Based on three critical assumptions, the MR method was developed: (1) The genetic variation must be closely related to the exposure in the MR analysis; (2) Genetic variation cannot be associated with confounding factors between exposure and outcome; (3) Exposure must be the mechanism through which genetic variables influence outcomes [21].

To assess whether potential mediators mediate between exposure and outcome, a two-step Mendelian randomization was used to assess the effect. The first step involved estimating the effect sizes of the exposure on lung function, lung cancer, asthma and mediators respectively. We use IVW as our primary method, which is characterized by regression without considering the presence of intercept terms and fitting with the reciprocal of the outcome variance as a weighting factor [22]. Additionally, we used MR-PRESSO, MR-Egger, and weighted median tests to estimate the effects. Subsequently, MVMR was used to determine the effect of each mediator on the outcome, taking into account how each instrument was genetically influenced [23].

**Fig. 1:**
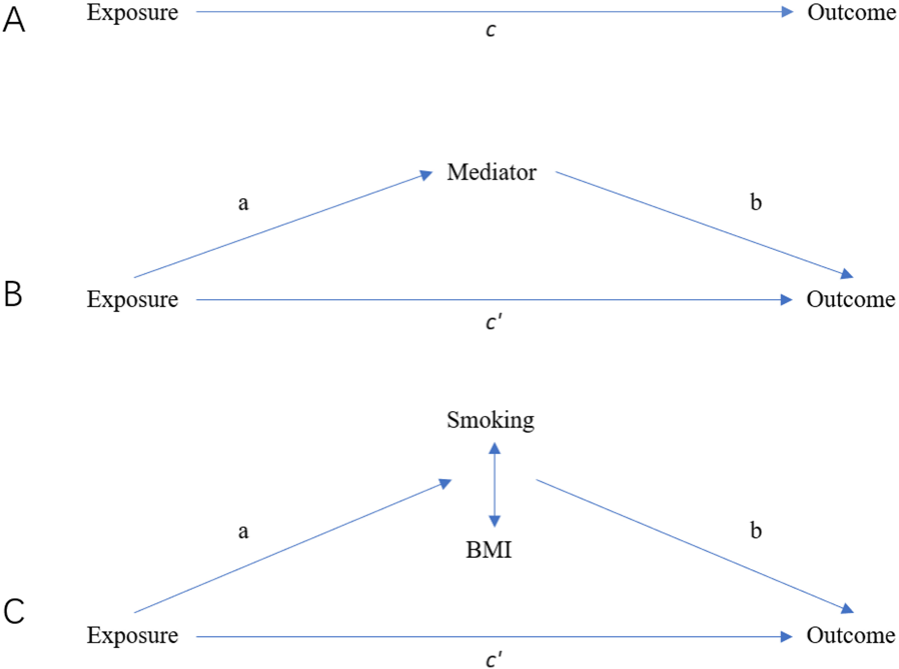
Diagrams illustrating associations examined in this study. **A** The total effect of exposure on outcome, c, was derived using univariable MR. **B** The total effect was decomposed into: (i) indirect effect using a two-step MR (where *a* is the total effect of exposure on mediator, *b* is the effect of mediator on outcome adjusting for exposure and the mediating effect is calculated using the product method (*a* × *b*)); (ii) direct effect (*c'* = *c* – *a* × *b*). **C** For mediation by both smoking and BMI combined (arrows represent their bidirectional causal relationship), the indirect effect was derived using the difference method (*c*–*c'*). Proportion mediated was the indirect effect divided by the total effect

Direct and indirect effects are both part of the total effect. Direct effect refers to the impact of the exposure factor on the outcome, independent of intermediary variables. Indirect effect, on the other hand, refers to the impact of the exposure factor on the outcome through intermediary variables [24]. The overall effect of EA on outcome was thus decomposed into two distinct components: (i) the indirect effect through each mediator individually, indicating the influence of education As a primary method for testing whether a mediated effect was present and its magnitude, we used the Sobel test (a × b in Fig. 1), and (ii) the direct effect of education on outcome after adjusting for each mediator (c' in Fig. 1) [25]. By using this statistical technique, we can explore complex relationships between variables and understand how intermediary variables impact exposure-outcome relationships.

To derive the indirect effect of combining multiple mediations, the difference method (c–c') is used, where c' indicates that multiple mediating factors are adjusted in the MVMR model. The delta method is used to calculate the confidence interval when the indirect effect is divided by the total effect (RMediation (shinyapps.io)), the proportion of the mediating effect can be quantified for one mediator or a combination of mediations. For each genetic instruments, we set *P* < 5 × 10^–8^ to selected genome-wide significant SNPs. To address the issue of linkage disequilibrium, we applied LD thresholds pairwise from the original GWAS for each mediator, with SNPs for each mediator adhering to an LD cut-off of r^2^ < 0.01 within a window of 1 MB.

### Sensitivity analysis

UVMR's IVW method can be examined for its robustness using two methods. Weighted medians are used in UVMR as well as Egger methods in MR Egger to assess the robustness of the IVW method, and Egger methods are used in MVMR to assess the robustness of the MVMR-IVW method. The MR-Egger method can determine whether horizontal pleiotropism exists in the instrumental variable to prevent violating the instrumental variable assumption. In addition, the Cochran's Q test is often used as an indicator of heterogeneity in meta-analysis, with a P-value less than 0.05 indicating the presence of heterogeneity in the study. An assessment of the strength of the genetic instrumental variables used in the study was conducted by using conditional F-statistics. A commonly used threshold for an "acceptable" F-statistic is 10, indicating that the instruments explain at least 10 times as much variance as the residual variance. However, this threshold may vary depending on the study design and sample size. In addition, we performed a “leave-one-out” sensitivity assessment to determine whether or not a certain SNP had too much influence on the results, and these SNPs were excluded from the MR analysis. Only when the IVW estimate agrees with at least one sensitivity analysis in direction and statistical significance, and there is no evidence of pleiotropy, is it considered to have a causal association.

The MR analyses were all performed using R (version 4.0.2) with the “TwoSampleMR” and “MRPRESSO” R package [26, 27].

### Patient and public involvement

The patient and public were not involved in the design or reporting of this study.

## Result

### Effect of education attainment on lung function, lung cancer and asthma

**Fig. 2:**
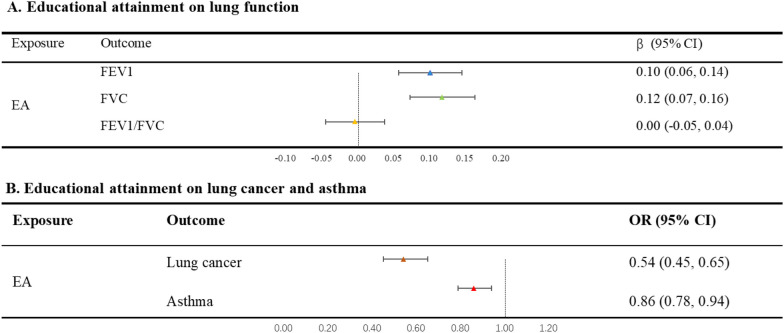
MR-estimated effects of educational attainment on each outcome separately, presented as β/OR with 95% CI. *EA* educational attainment, *FEV1* forced expiratory volume in one second, *FVC* forced vital capacity, *FEV1/FVC* forced expiratory volume in one second / forced vital capacity

The results of analyses found that increased genetically predicted EA was significantly related to enhanced FEV1 (β = 0.10, 95% CI 0.06, 0.14), improved FVC (β = 0.12, 95% CI 0.07, 0.16), and a less favorable FEV1/FVC ratio (β = -0.005, 95% CI − 0.05, 0.04). Furthermore, this heightened EA was also associated with a reduced risk of lung cancer (OR = 0.54, 95% CI 0.45, 0.65) and asthma (OR = 0.86, 95% CI 0.78, 0.94) (Fig. 2).

### Effect of education attainment on mediators

Table 2 shows the impact of education predicted by genetics on various mediators. A UVMR analysis revealed that for each extra 1-SD year of education are associated with lower BMI (IVW = − 0.16, 95% CI − 0.22, − 0.10), fewer cigarettes smoked per day (IVW = − 0.32, 95% CI − 0.40, − 0.24), and higher physical activity levels (IVW = 0.20, 95% CI 0.16, 0.23).

**Table 2:** Mendelian randomization analysis of the effect of educational attainment on mediators

Exposure	Outcome	Method	N of SNPs	β	SE	*P*
EA	BMI	MR Egger	106	− 0.1879	0.1476	0.2059
		Weighted Median	106	− 0.1601	0.0283	1.5467e−8
		IVW	106	− 0.1604	0.0314	3.3414e−07
	Physical activity	MR Egger	263	0.1425	0.0740	5.5370e−02
		Weighted Median	263	0.2020	0.0244	1.2701e−16
		IVW	263	0.1953	0.0186	8.2055e−26
	Cigarettes per day	MR Egger	242	− 0.1910	0.1619	2.3935e−01
		Weighted Median	242	− 0.2816	0.0500	1.7228e−08
		IVW	242	− 0.3218	0.0414	7.2237e−15

*EA* educational attainment, *BMI* body mass index, *IVW* inverse variance weighted, *MR* Mendelian randomization

### Effect of mediators on lung function, lung cancer and asthma after adjusting education attainment

**Fig. 3:**
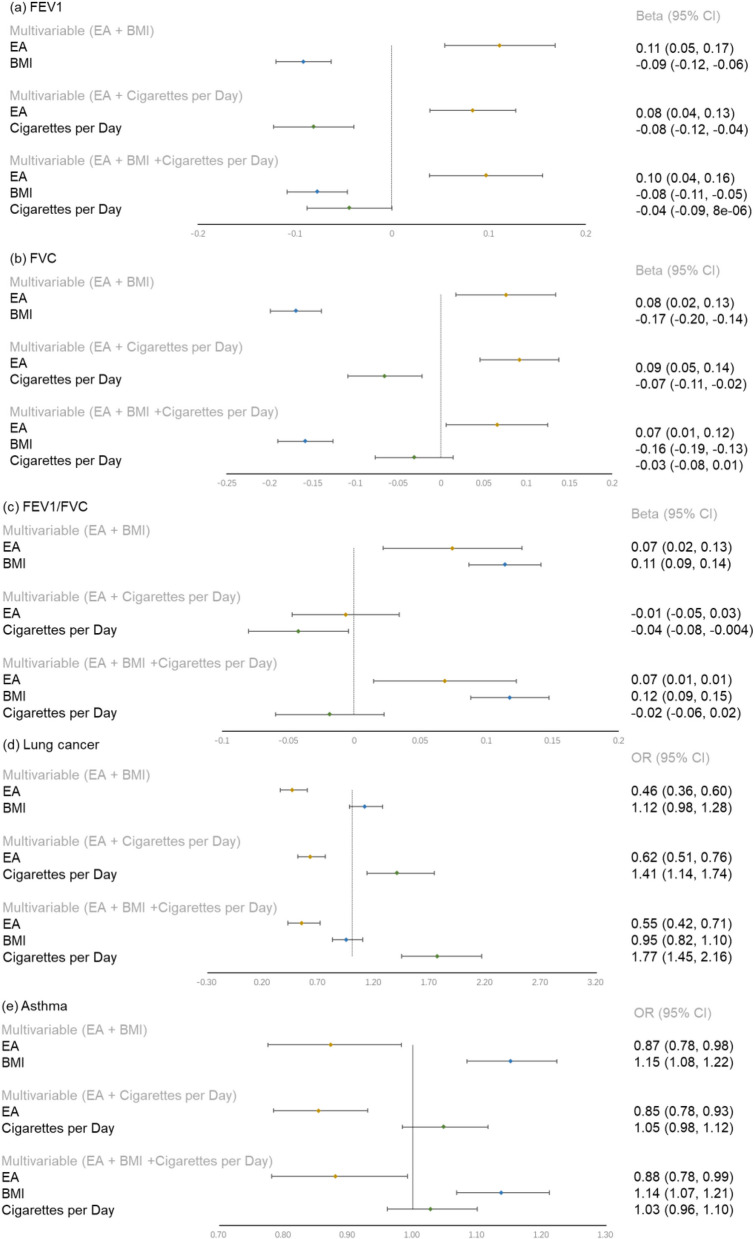
Effect of one standard deviation (SD) increase in exposure on outcome in multivariable models. EA, educational attainment; *BMI* body mass index, *FEV1* forced expiratory volume in one second, *FVC* forced vital capacity, *FEV1/FVC* forced expiratory volume in one second/forced vital capacity

According to Fig. 3, each mediator significantly predicted lung function and lung cancer after adjusting for EA. In this study, we excluded physical activity from our analysis because there was only one SNP available, which would lead to a large bias in the results. In the MVMR results, a 1-SD increase in BMI was associated with an increased risk of FEV1/FVC (β = 0.11, 95% CI 0.09, 0.14); lung cancer (OR = 1.12, 95% CI 0.98, 1.28); asthma (OR = 1.15, 95% CI 1.08, 1.22), and a 1-SD increase in genetically predicted cigarettes per day was associated with a higher risk of lung cancer (OR = 1.41, 95% CI 1.14, 1.74) and asthma (OR = 1.05, 95% CI 0.98,1.12). By contrast, each 1-SD unit higher BMI was associated with a reduced risk of FEV1 (β = − 0.09, 95% CI − 0.12, − 0.06) and FVC (β = − 0.17, 95% CI − 0.20, − 0.14), and a 1-SD lower genetically predicted cigarettes per day was associated with a decreased risk of FEV1 (β = − 0.08, 95% CI − 0.12, − 0.04), FVC (β = − 0.07, 95% CI − 0.11, − 0.02) and FEV1/FVC (β = − 0.04, 95% CI − 0.08, − 0.004).

### Mediating effect of mediators in the association between education attainment and lung function and respiratory diseases

In the MVMR analysis of the impact of EA to lung function through the consumption of cigarettes per day, the direct effect of EA on FEV1, FVC and FEV1/FVC was β = 0.08 (95% CI 0.04, 0.13), 0.09 (95% CI 0.05, 0.14) and β = − 0.01 (95% CI − 0.05, 0.03) after adjusting for the number of cigarettes smoked per day (Fig. 3). The direct effect of BMI on FEV1, FVC and FEV1/FVC was − 0.09 (95% CI − 0.12, − 0.06), − 0.17 (95% CI − 0.02, − 0.14) and 0.11 (95% CI 0.09, 0.14), respectively, after accounting for EA. The proportion mediated of FEV1, FVC and FEV1/FVC by BMI was 15%, 23% and 379%, respectively (Table 3).

**Table 3:** Estimates of the effect of educational attainment on outcomes explained by each mediator and by both combined

Mediating pathway	Indirect effect (a × b)	Proportion mediated (%)
*Educational attainment to FEV1*		
Via BMI	0.02 (0.01, 0.02)	0.15
Via cigarettes per day	0.03 (0.01, 0.04)	0.26
Via BMI + cigarettes per day	0.003	0.03
*Educational attainment to FVC*		
Via BMI	0.03 (0.02, 0.04)	0.23
Via cigarettes per day	0.02 (0.01, 0.04)	0.18
Via BMI + cigarettes per day	0.05	0.44
*Educational attainment to FEV1/FVC*		
Via BMI	− 0.02 (− 0.03, − 0.01)	3.79
Via cigarettes per day	0.01 (0.001, 0.03)	− 2.73
Via BMI + cigarettes per day	− 0.07	15.46
*Educational attainment to lung cancer*		
Via BMI	− 0.02 (− 0.04, 0.004)	0.03
Via cigarettes per day	− 0.11 (− 0.19, − 0.04)	0.18
Via BMI + cigarettes per day	− 0.02	0.03
*Educational attainment to asthma*		
Via BMI	− 0.02 (− 0.04, − 0.01)	0.15
Via cigarettes per day	− 0.02 (− 0.04, 0.005)	0.10
Via BMI + cigarettes per day	− 0.03	0.17

*BMI* body mass index, *FEV1* forced expiratory volume in one second, *FVC* forced vital capacity, *FEV1/FVC* forced expiratory volume in one second / forced vital capacity

The MVMR analysis revealed that the direct effect of EA on lung cancer and asthma through cigarette consumption per day was 0.62 (95% CI 0.51, 0.76) and 0.85 (95% CI 0.78, 0.93) after adjusting for cigarettes smoked per day (Fig. 3). The direct effect of cigarettes per day on lung cancer and asthma was OR = 1.41 (95% CI 1.14, 1.74) and OR = 1.05 (95% CI 0.98, 1.12) after accounting for EA. The proportion mediated of lung cancer and asthma by cigarettes per day was 18% and 10% (Table 3).

Both smoking and BMI were included in the FEV1 outcome MVMR model when considered simultaneously, effect sizes for EA (β = 0.10, 95% CI 0.04, 0.16), BMI (β = − 0.08, 95% CI − 0.11, − 0.05) and cigarettes per day (β = − 0.04, 95%CI − 0.09, 8e−06) (Fig. 3). Combined BMI and smoking mediated 44% of the effect of EA on FVC (Table 3). When BMI was the mediator, the effects of education on lung function and lung disease were shown in Fig. 3 and Table 3.

### MR sensitivity analyses

**Fig. 4:**
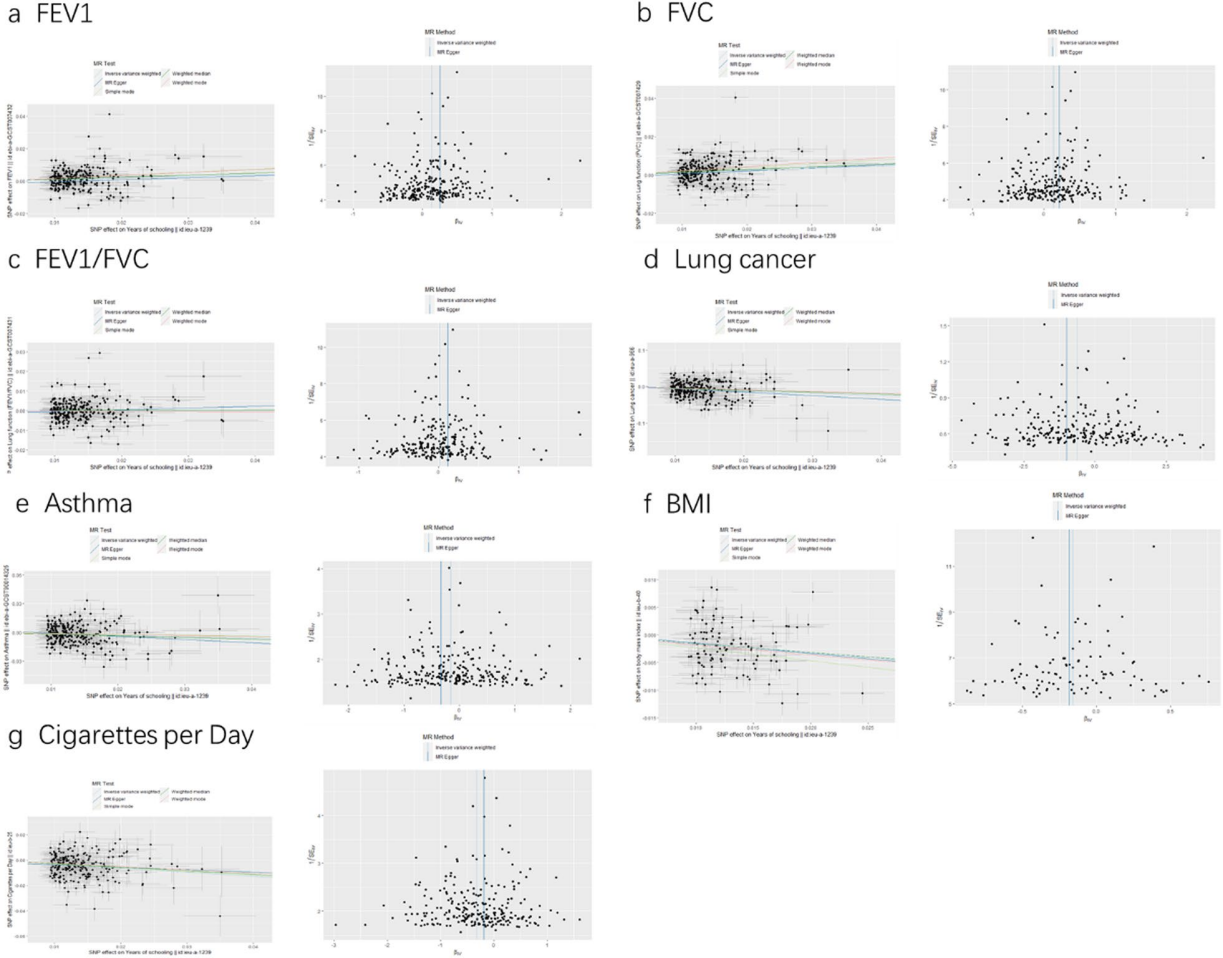
Mendelian randomization scatterplots and funnel plots of educational attainment to each mediator and outcome association. *BMI* body mass index, *FEV1* forced expiratory volume in one second, *FVC* forced vital capacity, *FEV1/FVC* forced expiratory volume in one second/forced vital capacity

According to the Cochran's Q test, the instrumental variables from education attainment to lung cancer did not show any heterogeneity, but there was heterogeneity in the other instrumental variables of the analysis which demonstrated a trend for the other instrumental variables (Table 4). In order to assess whether SNP has a horizontal pleiotropy, MR-Egger regression was used, which provided a valuable assessment of whether there was horizontal pleiotropy (Fig. 4). In the sensitivity analysis results, there was no significant evidence of directional pleiotropy (*P* > 0.05, Table 5). Furthermore, a further consistency between MR-weighted median and MR-IVW is in the direction of the distribution (Additional file 1: Table S1, Table 2). In reverse MR analyses between mediators and education attainment, the significant correlation between BMI and education attainment was found, but this reverse association could be due to horizontal pleiotropy (Egger intercept = − 0.0018; *P* = 0.0003). In terms of education attainment, Physical Activity and Cigarettes per day did not appear to have a causal effect (Additional file 1: Table S2). Moreover, leave-one-out analysis revealed that no SNP drove the results, and funnel plots were symmetrical (Fig. 4), indicating that the causal relationship has not been violated (Fig. 4). All SNPs have F-statistic ranging from 29.69 to 240.25. *F*-statistics > 10 considered suggestive of adequate instrument strength (Detailed information about SNPs is shown in Additional file 2: Table S3).

**Table 4:** MR heterogeneity test of the association of educational attainment with each outcome and mediator

	Q statistic	Q-*p*-value
*Educational attainment to FEV1*		
MR heterogeneity test (MR-Egger)	824.0855	1.15e−61
MR heterogeneity test (IVW)	824.3595	1.89e−61
*Educational attainment to FVC*		
MR heterogeneity test (MR-Egger)	916.0814	4.70e−75
MR heterogeneity test (IVW)	916.8741	6.69e−75
*Educational attainment to FEV1/FVC*		
MR heterogeneity test (MR-Egger)	721.9336	1.93e−46
MR heterogeneity test (IVW)	725.8082	9.23e−47
*Educational attainment to lung cancer*		
MR heterogeneity test (MR-Egger)	259.45	0.4454708
MR heterogeneity test (IVW)	260.4956	0.4447875
*Educational attainment to asthma*		
MR heterogeneity test (MR-Egger)	461.4252	2.63e−13
MR heterogeneity test (IVW)	463.3352	2.30e−13
*Educational attainment to BMI*		
MR heterogeneity test (MR-Egger)	502.4243	1.62e−53
MR heterogeneity test (IVW)	502.5996	3.34e−53
*Educational attainment to physical activity*		
MR heterogeneity test (MR-Egger)	371.1704	8.48e−06
MR heterogeneity test (IVW)	371.9444	9.04e−06
*Educational attainment to cigarettes per day*		
MR heterogeneity test (MR-Egger)	465.8943	1.25e−16
MR heterogeneity test (IVW)	467.2486	1.25e−16

*BMI* body mass index, *FEV1* forced expiratory volume in one second, *FVC* forced vital capacity, *FEV1/FVC* forced expiratory volume in one second/forced vital capacity, *IVW* inverse variance weighted, *MR* Mendelian randomization

**Table 5:** MR directional pleiotropy test (MR Egger) of the association of educational attainment with each outcome and mediator

Exposure	Outcome	Egger intercept	SE	*P* value
EA	FVC	− 0.0006	0.0012	0.6383
	FEV1	− 0.0013	0.0011	0.2441
	FEV1/FVC	− 0.0004	0.0012	0.2750
	Lung cancer	0.0052	0.0051	0.3098
	Asthma	0.0025	0.0024	0.300
	BMI	0.0004	0.0019	0.8493
	Physical activity	0.0007	0.0010	0.4613
	Cigarettes per day	− 0.0018	0.0022	0.4044

*BMI* body mass index, *FEV1* forced expiratory volume in one second, *FVC* forced vital capacity, *FEV1/FVC* forced expiratory volume in one second/forced vital capacity, *SE* standard error

## Discussion

In this MR study, the casual relationship between ET and respiratory functions and diseases was identified. To delve deeper into the mechanisms behind this association, we have identified three potential mediators from a pool of 23 modifiable risk factors. Our study findings reveal that education plays a crucial role in safeguarding lung function, preventing lung cancer, and mitigating the risk of asthma. An additional 4.2 years of schooling was associated with higher FEV1 and FVC values and lower lung cancer and asthma rates.

This is the first time that two-step MR analysis has been used to study the mediating relationship between EA and respiratory disease. Higher educational attainment is protective against respiratory disease, consistent with traditional observational findings. Actually, previous studies have shown that higher educational attainment has a protective effect on a range of health outcomes including lung cancer, artery stroke, type 2 diabetes. It is worth noting that this protective effect decreases as smoking and BMI are adjusted [28]. For example, smoking mediated 28% of the causal relationship between education and myocardial infarction, and BMI mediated 18% [29]. This shows that the implementation of public health measures to reduce smoking and obesity has wide-ranging benefits in preventing the occurrence of disease.

In this study, although the protective effect of education on respiratory diseases was verified, the mediating factor of choice explained only one quarter of the effect of education, leaving a significant portion unexplained. There are a number of other factors that may explain the remaining associations, including poverty, employment, diet, psychosocial factors, and access to healthcare [30–34]. However, since many of these factors are not heritable and cannot be obtained in GWAS, they cannot be included in this study.

In the UK, it has been proven that raising the age at which students leave school can have an impact on EA and lead to improvements in population health and a decrease in mortality rates[35]. Although EA has often been used as a proxy for socioeconomic status in previous studies, it is important to acknowledge that interventions solely targeting educational attainment may not offer an optimal solution for alleviating the burden of respiratory disease. In this study, a two-stage MR study was conducted to demonstrate that some risk factors mediate the relationship between EA and respiratory disease, and that these factors are more likely to change than EA.

In comparison to prior investigations, this study encompasses the following commendable attributes: (1) The study uses SNPs as genetic instruments can capture the impact of genetic variation on the phenotype or disease of interest. This approach effectively mitigates the confounding effects of reverse causality and errors. Due to allele random assignment at the time of conception, MR results that are insensitive to reverse causation. Additionally, using SNP as a tool variable can also improve the reliability and accuracy of MR analysis. (2) Exposure and outcome summary statistics in the study were obtained from the largest and most recent GWAS. (3) In order to improve the statistical power, a rigorous screening process was carried out for IVs (4) As part of the research process, multiple sensitivity analyses were performed in order to improve the results' accuracy. Furthermore, the MR analysis results align with those of observational studies, thereby reinforcing the robustness of the conclusions.

Notwithstanding the aforementioned strengths, this study is subject to some limitations that warrant consideration. Firstly, the GWAS used in the study exclusively featured on European populations. Thus, the generalization of results is not suitable for non-European people. Therefore, newer GWAS studies should focus on non-European populations. Secondly, given that EA has sex differences with respiratory diseases, associations and mediations may also differ between the sexes. However, as GWAS summary data were used, the effects of sex and age on outcomes could not be studied. A sex-stratified GWAS study may be used in future MR studies to address this issue. Thirdly, since lung cancer and asthma are binary variables, log-odds should be used in MR Analyses. The optimality of this approach is not achieved since the odds ratios do not collapse, i.e. marginal ORs are not equivalent to conditional odds ratios. Fourthly, the GWAS summary data used in this article comes from different repositories, in which case there is some heterogeneity between the data. This is inevitable, however, because when different data sources are selected, the bias of instrumental variables is reduced and the reliability of the results is improved. Finally, there is a possibility that GWAS results may be biased by sample overlap between studies.

## Conclusion

Elevated levels of EA may potentially exert a protective effect on respiratory diseases, with modifiable risk factors such as BMI and cigarettes per day mediating this relationship. Interventions to reduce smoking and adiposity may reduce much of this risk, which assumes even greater significance for individuals with respiratory disease. However, most of the remaining effects of EA on the relationship between respiratory disease remain unexplained. As such, there is a pressing need for enhanced preventive measures to address socioeconomic and educational disparities, as well as further research into other modifiable risk factors.

### Supplementary Information

**Additional file 1: Figure S1. **Overview of the process of identifying the mediators. **Table S1. **Mendelian randomization analysis of the effect of educational attainment on lung function and disease. **Table S2. **Reverse MR analysis of mediators to education attainment.

**Additional file 2: Table S3. **All instrumental variables used in Mendelian randomization analysis.

## Data Availability

The datasets analyzed in the current study are available in a public GWAS website (https://gwas.mrcieu.ac.uk/).
