# Supplementary material for: Association and mediation between educational attainment and respiratory diseases: a Mendelian randomization study
Source: Respir Res. 2024 Mar 6;25:115. doi: 10.1186/s12931-024-02722-4 (PMC10918882; doi:10.1186/s12931-024-02722-4)
Supplement: Supplementary file 1 — Additional file 1: Figure S1. Overview of the process of identifying the mediators. Table S1. Mendelian randomization analysis of the effect of educational attainment on lung function and disease. Table S2. Reverse MR analysis of mediators to education attainment. [file 12931_2024_2722_MOESM1_ESM.docx]

**Supplementary Online Content**

**Association and mediation between educational attainment and respiratory diseases: a Mendelian randomization study**

**Figure S1.** Overview of the process of identifying the mediators

**Table S1.** Mendelian randomization analysis of the effect of educational attainment on lung function and disease

**Table S2.** Reverse MR analysis of mediators to education attainment

**Table S3.** All instrumental variables used in Mendelian randomization analysis

**
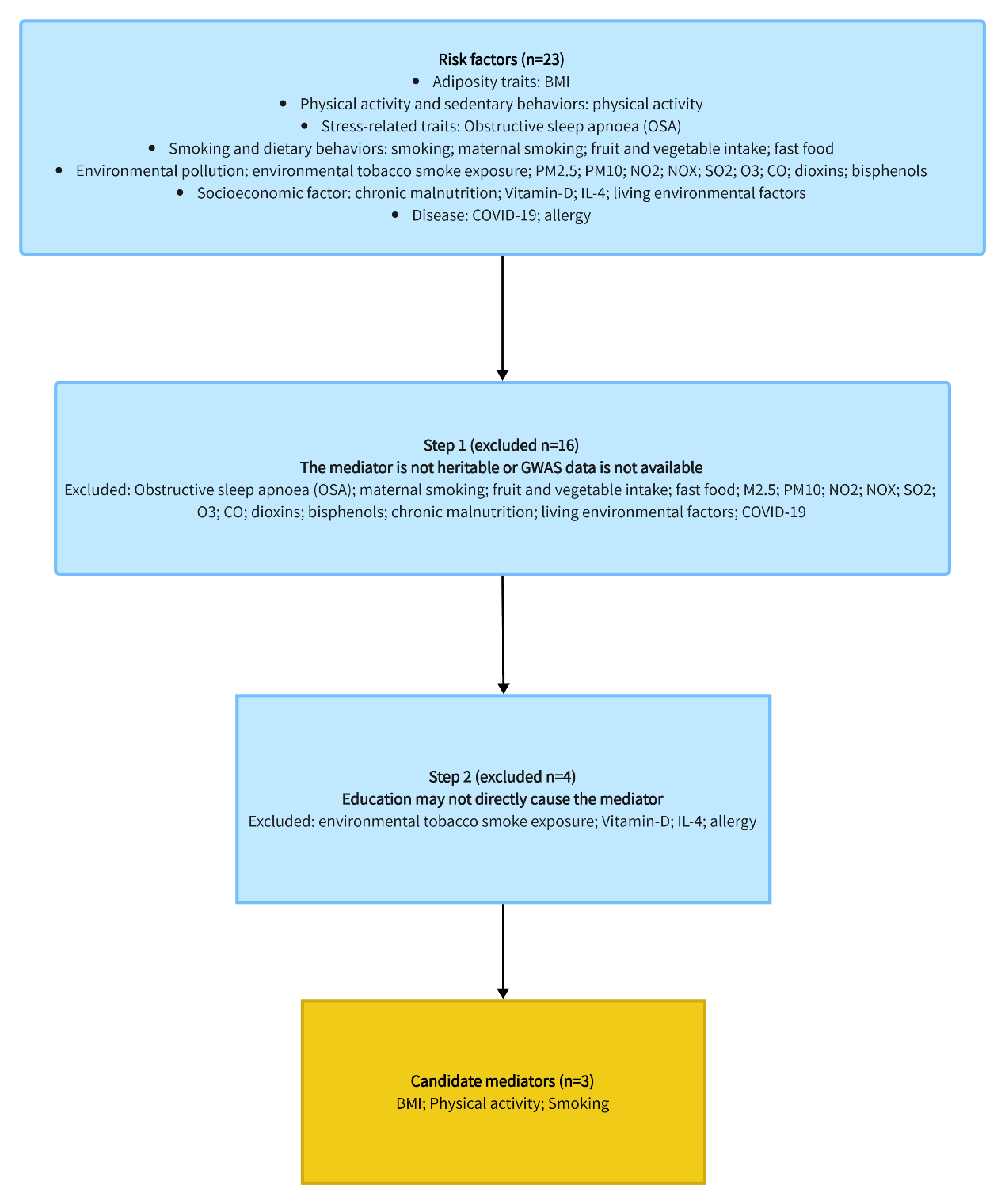
Figure S1. Overview of the process of identifying the mediators**

Abbreviations: BMI, body mass index; PM2.5, Particulate Matter 2.5; PM10, Particulate Matter 10; NO2, Nitrogen dioxide; NOX, NADPH Oxidases; SO2, Sulfur dioxide; O3, Ozone; CO, Carbon Monoxide; IL-4, Interleukin 4; COVID-19, Corona Virus Disease 2019.

**Table S1. Mendelian randomization analysis of the effect of educational attainment on lung function and disease**

| Exposure | | Outcome | Method | N of SNPs | | β | SE | *P* |
| --- | --- | --- | --- | --- | --- | --- | --- | --- |
| EA | FEV1 | MR Egger | | 255 | 0.1256 | 0.0915 | 0.1709 |  |
|  |  | Weighted Median | | 255 | 0.1244 | 0.0221 | 1.7024e-08 |  |
|  |  | IVW | | 255 | 0.0999 | 0.0223 | 7.4880E-06 |  |
|  | FVC | MR Egger | | 258 | 0.1586 | 0.0919 | 8.5622e-02 |  |
|  |  | Weighted Median | | 258 | 0.1399 | 0.0226 | 6.3358e-10 |  |
|  |  | IVW | | 258 | 0.1167 | 0.0232 | 4.6743e-07 |  |
|  | FEV1/FVC | MR Egger | | 256 | 0.0892 | 0.0832 | 0.2845 |  |
|  |  | Weighted Median | | 256 | 0.0084 | 0.0220 | 0.7025 |  |
|  |  | IVW | | 256 | -0.0048 | 0.0210 | 0.8210 |  |
|  | Lung cancer | MR Egger | | 259 | -0.9932 | 0.3800 | 9.4962e-03 |  |
|  |  | Weighted Median | | 259 | -0.5150 | 0.1445 | 3.6629e-04 |  |
|  |  | IVW | | 259 | -0.6185 | 0.0943 | 5.3071e-11 |  |
|  | asthma | MR Egger | | 263 | -0.3334 | 0.1781 | 0.06225 |  |
|  |  | Weighted Median | | 263 | -0.1735 | 0.0534 | 0.0012 |  |
|  |  | IVW | | 263 | -0.1543 | 0.0448 | 0.0006 |  |

Abbreviations: FEV1, forced expiratory volume in one second; FVC, forced vital capacity; FEV1/FVC, forced expiratory volume in one second／forced vital capacity; IVW, inverse variance weighted; MR, Mendelian randomization; SE, standard error.

**Table S2. Reverse MR analysis of mediators to education attainment**

| Exposure | Outcome | Method | MR results | | | Heterogeneity test | | pleiotropy | |
| --- | --- | --- | --- | --- | --- | --- | --- | --- | --- |
|  |  |  | β | SE | *P* | Q | *P* | Egger intercept | *P* |
| BMI | Education attainment | MR Egger | -0.0108 | 0.0302 | 7.2024e-01 | 1716.322 | 3.4128e-156 | -0.0018 | 0.0003 |
|  |  | Weighted Median | -0.0977 | 0.0125 | 4.9131e-15 | NA | NA |  |  |
|  |  | IVW | -0.1429 | 0.0144 | 3.2630e-23 | 1769.652 | 1.0781e-164 |  |  |
| Physical activity |  | MR Egger | 2.4873 | 0.8983 | 0.2206 | 0.1343 | 0.7140 | -0.0420 | 0.2318 |
|  |  | Weighted Median | 0.1055 | 0.0911 | 0.2466 | NA | NA |  |  |
|  |  | IVW | 0.1355 | 0.1067 | 0.2041 | 7.0164 | 0.0300 |  |  |
| Cigarettes per day |  | MR Egger | 0.0377 | 0.0159 | 0.0330 | 25.4123 | 3.0708e-02 | -0.0049 | 0.0009 |
|  |  | Weighted Median | -0.0003 | 0.0087 | 0.9740 | NA | NA |  |  |
|  |  | IVW | -0.0167 | 0.0135 | 0.2156 | 57.5578 | 6.5979e-07 |  |  |

Abbreviations: BMI, body mass index; IVW, inverse variance weighted; MR, Mendelian randomization; SE, standard error.
